# Retraction Note to: Downregulating lncRNA NEAT1 induces proliferation and represses apoptosis of ovarian granulosa cells in polycystic ovary syndrome via microRNA-381/IGF1 axis

**DOI:** 10.1186/s12929-022-00805-2

**Published:** 2022-03-30

**Authors:** Jingran Zhen, Jiangli Li, Xia Li, Xue Wang, Yaling Xiao, Zhengyi Sun, Qi Yu

**Affiliations:** 1 Department of Gynecological Endocrinology and Reproduction Center, Peking Union Medical College Hospital, Chinese Academy of Medical Sciences, 41 Damucang Hutong, Xicheng, Beijing, China; 2 Department of Obstetrics and Gynecology, Zhongguancun Hospital, Beijing, 100080 China; 3 Community Health Service Center, Beijing Forestry University, Beijing, 100053 China

## Retraction to: J Biomed Sci (2021) 28:53 10.1186/s12929-021-00749-z

The Editor-in-Chief has retracted this Article. Concerns were raised about the authenticity of the western blot data. The Authors were unable to provide uncropped images of the original gels. Additionally, there are fundamental flaws in the flow cytometry apoptosis assay methodology. The Editor-in-Chief therefore no longer has confidence in the data and conclusions of this study.

Zhengyi Sun agrees to this retraction. Jingran Zhen has not explicitly stated whether they agree to this retraction notice. Qi Yu does not agree to this retraction. Jiangli Li, Xia Li, Xue Wang and Yaling Xiao have not responded to any correspondence from the editor about this retraction.

